# The causal relationship between inflammatory bowel disease and primary biliary cholangitis: A bidirectional two-sample Mendelian randomization study

**DOI:** 10.1097/MD.0000000000046666

**Published:** 2025-12-19

**Authors:** Zongchi Chen, Wenyuan Hong, Xinxia Yang, Weitao Hu, Taiyong Fang

**Affiliations:** a Department of Gastroenterology, The Second Affiliated Hospital of Fujian Medical University, Quanzhou, Fujian, P.R. China; b Endoscopic Center, Anxi Maternal and Child Health Hospital, An Xi, Fujian, P.R. China.

**Keywords:** Crohn disease, inflammatory bowel disease, Mendelian randomization study, primary biliary cholangitis, ulcerative colitis

## Abstract

The cause-and-effect relationship between inflammatory bowel disease (IBD) and primary biliary cholangitis (PBC) has not been fully elucidated. Thus, this study explored their causal relationship through bidirectional 2-sample Mendelian randomization (MR). Genome-wide association studies were used to obtain data. Single-nucleotide polymorphisms (SNPs) closely related to IBD (including ulcerative colitis [UC] and Crohn disease [CD]) and PBC were screened, and the methods used included inverse variance weighted and the weighted median method. MR analysis was performed using maximum likelihood and MR-Egger. Sensitivity analysis was performed to identify heterogeneity and horizontal pleiotropy, thereby assessing the reliability of the results. Gene-predicted IBD (odds ratio [OR]: 1.20, 95% confidence interval [CI]: 1.09–1.31; *P* < .001) and CD (OR: 1.16, 95% CI: 1.08–1.25; *P* < .001) were positively associated with PBC. However, we failed to detect statistical evidence of correlation between UC and PBC (OR: 0.90, 95% CI: 0.80–1.01, *P* = .08). Reverse analysis demonstrated a causal relationship between genetic predisposition to PBC and increased risk of IBD, but no reverse causation for CD and UC. This MR study elucidates a bidirectional association between IBD and PBC. Specifically, CD exerted a potential positive causal effect on PBC, while no reverse causation was observed. Conversely, UC exhibited no causal relationship with PBC.

## 1. Introduction

Inflammatory bowel disease (IBD) is a chronic, idiopathic inflammatory condition of the bowel, characterized by chronic inflammation of the bowel, recurring episodes, and an elusive etiology. It encompasses conditions such as ulcerative colitis (UC) and Crohn disease (CD). At present, IBD is becoming a global health concern, with a prevalence rate exceeding 0.3% and a rising incidence in several countries.^[1]^ IBD has several extraintestinal manifestations, collectively known as extraintestinal manifestations, in addition to its primary expression as gastrointestinal inflammation.^[2]^ Studies have shown that 50% of patients with IBD will exhibit manifestations of hepatobiliary diseases; primary biliary cholangitis (PBC) is one such manifestation.^[3]^

PBC is a chronic, immune-mediated liver disease. It is marked by increasing cholestasis. According to the January 2022 Orphanet report on the prevalence of rare diseases, its average annual incidence rate is 3 cases per 100,000 individuals per year. With a prevalence of only 21 cases per 100,000 people, PBC is considered a rare disease.^[4]^ However, recent studies have identified an upward trend in the past few decades.^[5–8]^ In addition to abnormal liver function indicators or itchy skin, PBC can also occur as a result of extra-hepatic diseases of the immune system, such as IBD, Hashimoto’s thyroiditis, and osteoarthritis rheumatica, and patients with both IBD and PBC have also been reported.^[3,9,10]^ Liberal et al^[11]^ expressed the same standpoint in their review.

Although the pathogenesis of IBD remains unclear, it is likely to be the result of a complex interaction between genetic and environmental factors. There is increasing evidence that familial genetic predisposition and the presence of other autoimmune diseases may be closely related to the pathogenesis of PBC.^[12–16]^ Therefore, while a specific relationship may exist between the pathogenesis of these 2 diseases, it remains to be elucidated.

Significant clues for the diagnosis and treatment of IBD and PBC can be gained by studying the causal relationship between the 2 diseases. While randomized controlled studies represent the most robust method for inferring causal relationships, their practical implementation can be hindered by logistical and ethical constraints inherent in real-life settings. Based on observational epidemiological studies, we conducted a Mendelian randomization (MR) analysis to explore the possible role of causality between IBD and PBC, with the goal of uncovering any underlying associations between the 2 conditions through rigorous genetic investigation.

Unlike traditional observational studies, MR can mitigate the impacts of potential confounders and reverse causation.^[17–19]^ This is because MR analysis uses genetic variation as an instrumental variable for the detection and quantification of causal relationships.^[20]^ These genetic alleles, associated with the exposure of interest, are randomly assigned at conception, free from the influence of personal lifestyle and immune to environmental factors or disease-related interference.^[21,22]^ Thus, this study used a 2-sample MR approach and comprehensive statistical analysis to investigate the cause-and-effect relationship between IBD (UC/CD) and PBC.

## 2. Materials and methods

### 2.1. Research design

**Figure 1.**
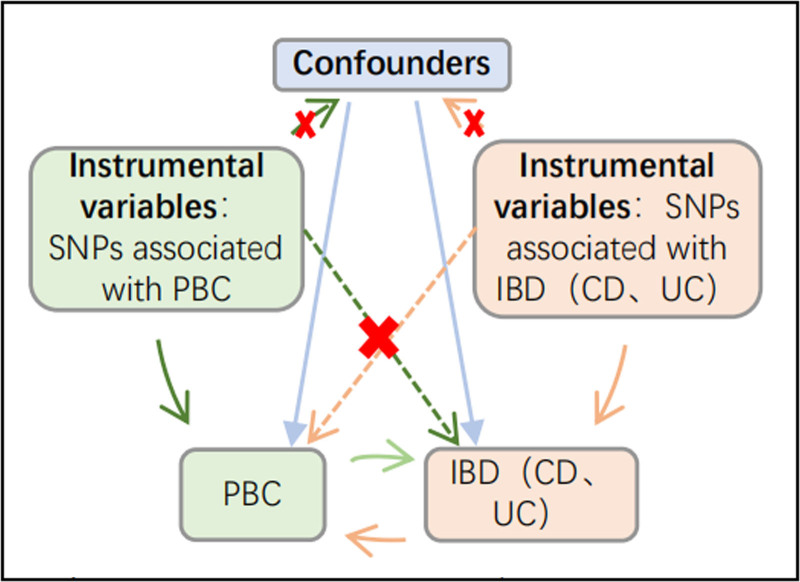
Study design of the bidirectional Mendelian randomization between IBD (CD, UC) and PBC. The brown solid lines represent the association between the instrumental variables and exposure, as well as the association between exposure and outcome. The green solid lines represent the association of reverse causality. The figure illustrates 3 basic assumptions of Mendelian randomization: the variant is associated with exposure; the genetic variants are independent of confounders between exposure and outcomes; and the genetic variants only influence the outcome via exposure. CD = Crohn disease, IBD = inflammatory bowel disease, PBC = primary biliary cholangitis, UC = ulcerative colitis.

This study is a bidirectional 2-sample MR study. The 3 following assumptions need to be fulfilled: genetic variables are strongly related to exposure; genetic factors are unrelated to confounders between exposure and outcome^[23]^; and genetic factors do not directly affect outcomes, but indirectly affect outcomes through exposure.^[24]^ Based on many large-sample genome-wide association studies (GWASs),^[1]^ we selected single-nucleotide polymorphisms (SNPs) that are closely related to IBD and PBC as instrumental variables. The impact of instrumental variables on both exposure and outcomes is established based on data collected from 2 separate sample groups, each with its own unique characteristics and parameters that contribute to the overall analysis (Fig. 1). All research data used was obtained from published studies that were reviewed by appropriate ethics committees. No new ethics approval was required.

### 2.2. Data source

SNPs were screened from published GWAS databases related to IBD and PBC (Table 1). IBD data statistics were obtained from the genetic association data of the International Inflammatory Bowel Disease Genetics Consortium, from the European population. These included 31,665 studies and 33,977 control cases. The 27,432 UC cases included 6968 studies and 20,464 control cases. The 51,874 CD cases included 17,897 studies and 33,977 control cases. PBC data statistics were obtained from the genetic association data of the UK Biobank, including 2861 studies and 8514 control cases. To minimize potential sample overlap, we ensured that these datasets were obtained from independent cohorts. The inverse variance weighted (IVW) random effects model was used for quality control of the correlation between the GWAS and the population.

**Table 1:** Summary of GWAS data.

	Dataset	PMID	Case (N)	Control (N)	Sample size	Population	Consortium	Year
IBD	ieu-a-294	26192919	31,665	33,977	65,642	European	IIBDGC	2015
CD	ieu-a-12	26192919	17,897	33,977	51,874	European	IIBDGC	2015
UC	ieu-a-32	26192919	6968	20,464	27,432	European	IIBDGC	2015
PBC	Ebi-a-GCST005581	22961000	2861	8514	11,375	European	NA	2012

GWAS = genome-wide association study, IBD = inflammatory bowel disease, PBC = primary biliary cholangitis, SNPs = single-nucleotide polymorphisms, UC = ulcerative colitis.

### 2.3. SNP selection

We summarized the previously collected SNPs and filtered the data and removed linkage disequilibrium (*P* < 5 × 10^-8^, *R*^2^ < 0.001).^[1]^ Verification of instrumental variable strength using *F*-statistic values (*F*>10). The *F* value was calculated using *F* = $\left(\frac{R^{2}}{1-\;\;\;R^{2}}\right)$$\left(\frac{n-k-1}{k}\right)$ ($R^{2}=2\times \left(1-MAF\right)\times MAF\times \beta^{2}$, β is the SNP used as the instrumental variable for exposure, MAF stands for minimum allele frequency, N is the simple size of the total sample size, is the number of instrumental variables). We then merged the exposure data with the outcome data and removed palindromic SNPs. We selected instrumental variables that strongly correlated with exposure, and finally used the MR-Steiger directional test to filter SNP data with reverse causality.^[25]^ The remaining SNP was the final instrumental variable referring to exposure (Table 2).

**Table 2:** Instrumental variable.

Exposure	Outcome	SNPs									
IBD	PBC	rs10142466	rs1182188	rs13204742	rs1990760	rs272882	rs4703855	rs62434177	rs7015630	rs7848647	
		rs10758669	rs12103	rs13407913	rs2024092	rs2836883	rs4743820	rs6456426	rs7194886	rs79980175	
		rs10761659	rs12318183	rs1363907	rs2050392	rs2847278	rs4976646	rs648541	rs7240004	rs913678	
		rs10800309	rs1250566	rs1388585	rs2143178	rs3024493	rs516246	rs6500315	rs7253253	rs9264942	
		rs11152949	rs12585310	rs140143	rs2153283	rs34779708	rs55808324	rs6561151	rs72634258	rs941823	
		rs11185982	rs1267499	rs1517352	rs2270395	rs34804116	rs559928	rs6584281	rs72924296	rs9457247	
		rs11230563	rs12718244	rs17293632	rs2328546	rs35256947	rs56167332	rs6651252	rs744166	rs974801	
		rs11236797	rs12722515	rs17694108	rs2395022	rs3776414	rs6058869	rs6708373	rs7523442	rs9836291	
		rs11677953	rs1292053	rs17780256	rs2497318	rs3801835	rs6062496	rs6740462	rs7608910	rs9889296	
		rs11691685	rs1297258	rs181826	rs2538470	rs3853824	rs6111031	rs6745185	rs7711427		
		rs11793497	rs13107612	rs1847472	rs259964	rs4692386	rs62037363	rs7011507	rs780094		
UC	PBC	rs10737481	rs10737481	rs114152040	rs1359946	rs1801274	rs3829111	rs4676410	rs483905		
		rs56167332	rs6017342	rs7523335	rs7911680	rs9977672					
CD	PBC	rs10758669	rs1250573	rs1456896	rs212388	rs26528	rs36016881	rs61839660	rs6908425	rs76906269	rs9457247
		rs10798069	rs1267501	rs1517352	rs2153283	rs3024505	rs3776414	rs640466	rs7015630	rs7711427	rs9491892
		rs10800309	rs12694846	rs17129991	rs2227551	rs303429	rs3801810	rs6456426	rs7085798	rs7786444	rs9494844
		rs11152949	rs1292053	rs17293632	rs2270395	rs3129871	rs3853824	rs6500315	rs71624119	rs77981966	rs9889296
		rs11159833	rs12949918	rs17391694	rs2284553	rs3197999	rs438475	rs6561151	rs7194886	rs780094	
		rs11167518	rs1297258	rs17622378	rs2395022	rs34592089	rs4703855	rs6651252	rs7236492	rs7848647	
		rs11185982	rs13001325	rs17694108	rs2413583	rs34779708	rs516246	rs6738394	rs72727394	rs7969592	
		rs11236797	rs13407913	rs181826	rs2538470	rs34804116	rs56163845	rs6738490	rs7438704	rs79980175	
		rs11691685	rs1363907	rs1847472	rs259964	rs35164067	rs6062496	rs6740462	rs7517847	rs915286	
		rs11793497	rs140143	rs2024092	rs2641348	rs35320439	rs6111031	rs6827756	rs7608910	rs9264942	
PBC	IBD	rs10931468	rs12708716	rs17122453	rs2523882	rs34725611	rs35188261	rs6871748	rs7665090	rs7775055	rs911263
PBC	UC	rs10931468	rs11117431	rs12134279	rs12708716	rs1646019	rs17122453	rs1800693	rs2293370	rs34725611	rs35188261
		rs522127	rs6871748	rs79513546	rs911263						
PBC	CD	rs12708716	rs17122453	rs2293370	rs2523882	rs35188261	rs522127	rs7665090	rs911263		

IBD = inflammatory bowel disease, PBC = primary biliary cholangitis, SNPs = single-nucleotide polymorphisms, UC = ulcerative colitis.

### 2.4. Statistical analysis

We applie9d MR-Steiger analysis to test the direction of potential causal relationships between each extracted SNP. We used IVW as the primary MR method to establish the link between IBD (UC and CD) and PBC. MR-Egger analyses, weighted median (WM), and maximum likelihood (ML) were used as supplementary IVW MR.IVW is a weighted average of effect sizes, using inverse variance as weight to summarize effect sizes of each study across multiple independent studies; it is a standard MR method used to summarize data^[26]^ and was therefore used as the main result. As IVW analysis assumes that tools can only affect results through the exposure of interest,^[27]^ which may be affected by invalid cooccurrence bias or pleiotropy, we used WM with MR-Egger. This can provide more robust estimates and supplement IVW estimates but at the cost of lower effect sizes from these methods.^[28]^ The MR-Egger regression provides MR estimates and adjusts for horizontal pleiotropy through its intercept test. The MR-pleiotropic residual sum and outliers (MR-PRESSO) method can detect SNP outliers with pleiotropic effects and provides estimates that are identical to IVW after removing these outliers. The reliability of the results obtained from the analysis was verified using MR-pleiotropic residual sum and outliers (MR-PRESSO).In the weighted median method, when ≥ 50% of SNPs are valid IVs, consistent causal estimates can be generated and reliable conclusions can be drawn. The maximum likelihood method can combine data from multiple genetic variants into a single causal estimate, and when the same assumptions are met, its standard error is smaller. The maximum likelihood method can complement the MR-EGGER regression method.

For sensitivity analysis, we first used the leave-one-out meta-analysis algorithm to eliminate SNP data that strongly correlated with the outcome; we then reanalyzed the cause-effect relationship between the instrumental variable and outcome. Simultaneously, the 2-sample MR R package was used to conduct Cochran Q test^[1]^ on SNPs that conformed to Mendelian assumptions; this was used to evaluate the heterogeneity between individual genetic variants. Additionally, the MR-Egger intercept test and funnel plot were used to perform sensitivity analysis on the results.

A 2-sided *P*-value of < .017 (Bonferroni correction) was found to be statistically significant. All statistical analyses were performed with R 3.4.2 utilizing the package “two-sample MR.”

## 3. Results

### 3.1. Causality of IBD on PBC

**Figure 2.**
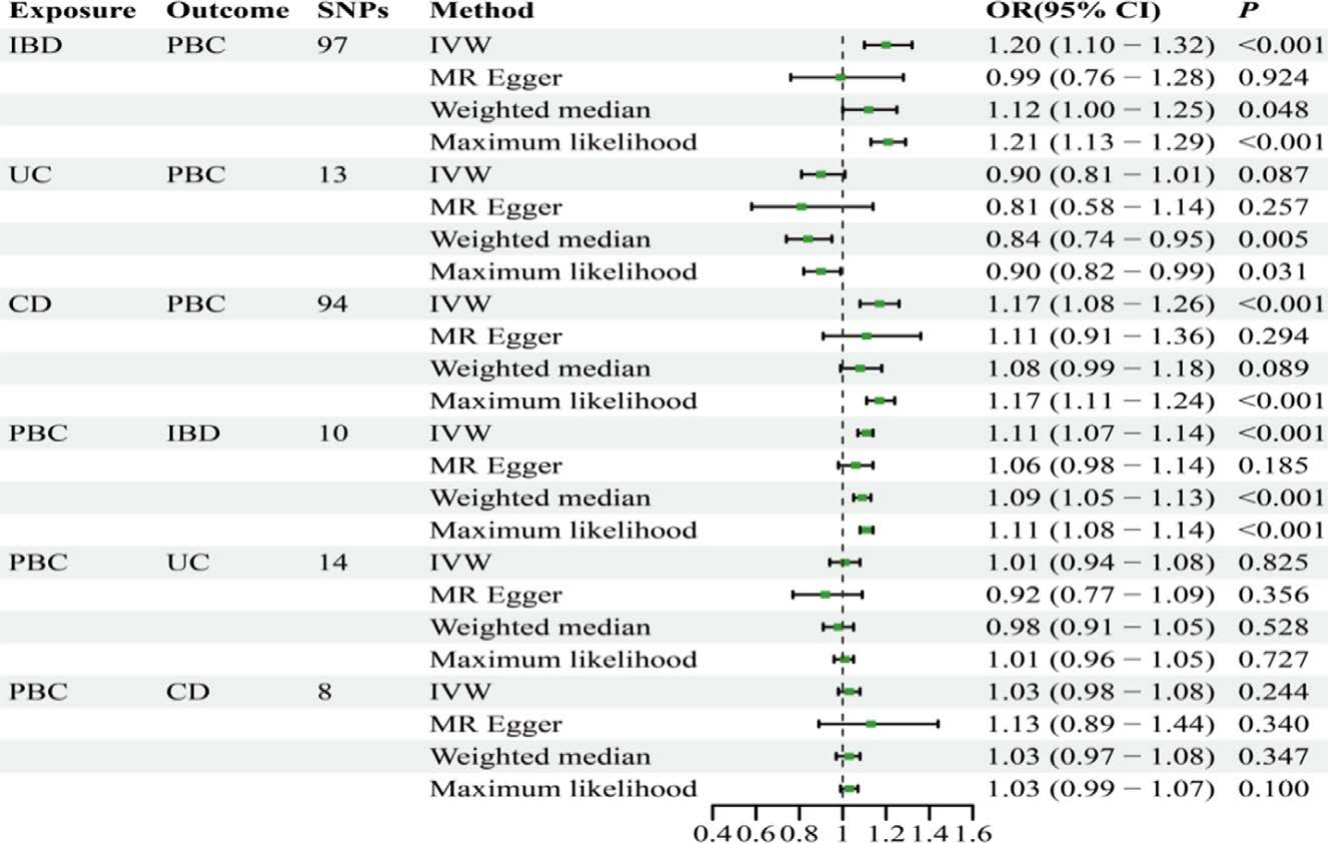
MR estimates from different methods assessing the causal effects between UC, CD, IBD and PBC. CD = Crohn disease, IBD = inflammatory bowel disease, PBC = primary biliary cholangitis, UC = ulcerative colitis.

**Figure 3.**
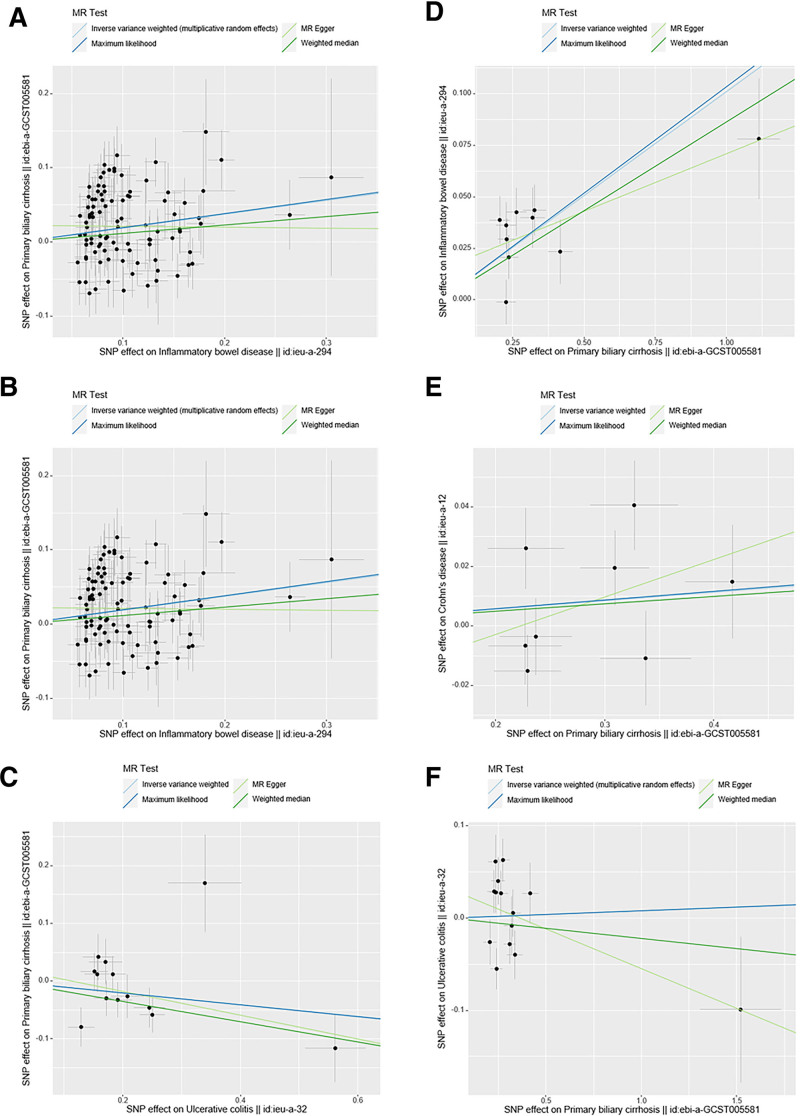
Scatter plots of the genetic causal associations between IBD, UC, CD, and PBC using different MR methods. (A) IBD against PBC; (B) CD against PBC; (C) UC against PBC; (D) PBC against IBD; (E) PBC against CD; (F) PBC against UC. The slopes of the line represent the causal association for different methods. The light green line represents the MREgger, the dark green line represents the WM estimate, the light blue line represents the IVW estimate, and the dark blue line represents the ML estimate. CD = Crohn disease, IBD = inflammatory bowel disease, IVW = inverse variance weighted, ML = maximum likelihood, MR = Mendelian randomization, PBC = primary biliary cholangitis, UC = ulcerative colitis, WM = weighted median.

**Figure 4.**
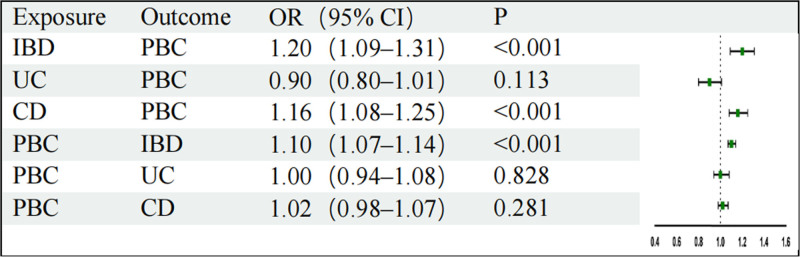
MR-PRESSO assessing the causal effects between UC, CD, IBD, and PBC. CD = Crohn disease, IBD = inflammatory bowel disease, PBC = primary biliary cholangitis, UC = ulcerative colitis.

**Figure 5.**
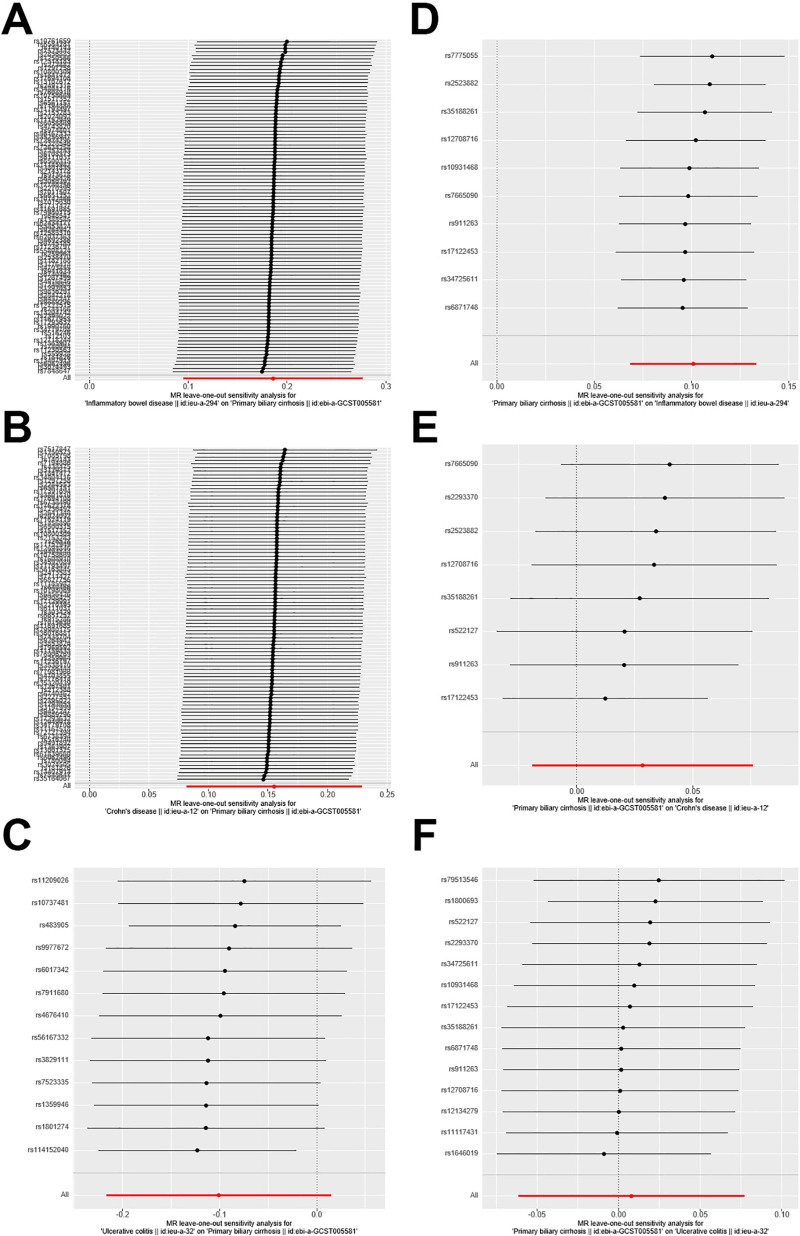
Forest plots of SNPs associated with UC, CD, and PBC. (A) IBD against PBC; (B) CD against PBC; (C) UC against PBC; (D) PBC against IBD; (E) PBC against CD; (F) PBC against UC. CD = Crohn disease, IBD = inflammatory bowel disease, PBC = primary biliary cholangitis, SNP = single-nucleotide polymorphism, UC = ulcerative colitis.

**Figure 6.**
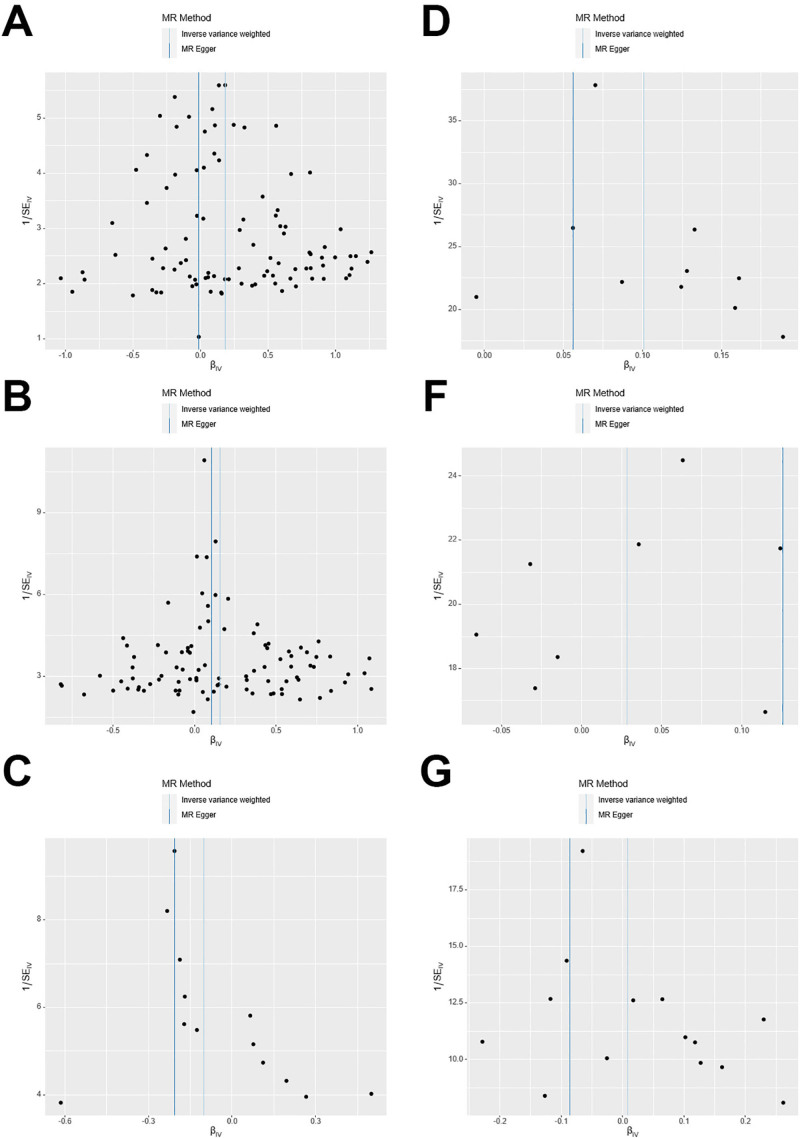
Causal relationships between UC, CD, and PBC in funnel plots. (A) IBD against PBC; (B) CD against PBC; (C) UC against PBC; (D) PBC against IBD; (E) PBC against CD; (F) PBC against UC. CD = Crohn disease, IBD = inflammatory bowel disease, PBC = primary biliary cholangitis, UC = ulcerative colitis.

Based on the above selection criteria, 97 SNPs were finally included. We conducted IVW analysis and found that IBD had a causal effect on PBC (odds ratio [OR]: 1.20, 95% confidence interval [CI]: 1.09–1.31; Fig. 2), which is statistically significant. WM and ML analyses revealed a causal effect in the same direction (Fig. 3). Additionally, using MR-PRESSO as a supplement to IVW, we obtained similar results with *P* < .05, which strengthened the robustness of the results (Fig. 4). Directionality tests conducted using Steiger-MR confirmed our estimates of potential causal directions (*P* < .001), confirming the stability of causal effect estimates. In the sensitivity analysis, MR-Egger intercept analysis (intercept = 0.02; *P* = .11) revealed that no horizontal pleiotropy occurred (Table 3). Although Cochran Q test resulted in *P* = 1.89E-07 and heterogeneity was observed (Table 3), our study used IVW as a random effects model, which can correct the heterogeneity of the results. Leave-one-out meta-analysis showed no influence from a single SNP (Fig. 5A). Visual inspection of the funnel plot of the causal relationship between IBD and PBC showed that all point estimates were symmetric (Fig. 6A).

**Table 3:** Sensitivity analysis.

Exposure	Outcome	MR-presso Global Test		Cochran Q
		intercept	*p*-value	*p*-value
IBD	PBC	0.02	0.11	1.89 × 10^-7^
UC	PBC	0.02	0.52	0.08
CD	PBC	0.01	0.60	1.66 × 10^-7^
PBC	IBD	0.01	0.23	0.09
PBC	UC	0.03	0.27	2.34 × 10^-3^
PBC	CD	-0.02	0.44	0.06

IBD = inflammatory bowel disease, MR-PRESSO = MR-pleiotropic residual sum and outliers, PBC = primary biliary cholangitis, SNPs = single-nucleotide polymorphisms, UC = ulcerative colitis.

### 3.2. Causal relationship between CD and PBC

Based on the above selection criteria, 94 SNPs were finally included; IVW analysis revealed that gene-predicted CD positively correlated with PBC (OR: 1.16, 95% CI: 1.08–1.25; Fig. 2). Using MR-PRESSO as a supplement to IVW, we obtained similar results, which strengthened the robustness of the results (Fig. 4). In the sensitivity analysis, no horizontal pleiotropy was observed using MR-Egger intercept analysis (intercept = 0.01; *P* = .60; Table 1). Although heterogeneity was observed through Cochran Q test, it did not impact the results of the IVW analysis, and the results obtained remained reliable. Leave-one-out meta-analysis revealed that the MR results were not affected by a single SNP (Fig. 5B), and the MR estimate was reasonable. For the funnel plot of the causal relationship between CD and PBC, all point estimates were visually inspected to be symmetric (Fig. 6B).

### 3.3. Causality of UC on PBC

After the above screening, 13 SNPs were included; the IVW results revealed (OR: 0.90, 95% CI: 0.80–1.01; *P* = .08) that UC has no causal effect on PBC. MR-Egger and ML analyses obtained similar causal effect estimates. No significant heterogeneity was found using Cochran Q test (Table 3). Leave-one-out meta-analysis showed that the overall estimate was not affected by individual SNPs. Additionally, no horizontal pleiotropy was observed in the MR-Egger intercept analysis (intercept: 0.02; *P* = .52; Table 3).

### 3.4. Causality of PBC on IBD

After selecting 10 SNPs, IVW analysis was performed, demonstrating that PBC has a causal effect on IBD (OR: 1.10, 95% CI: 1.07–1.14; *P* = 1.10E-09); this finding was statistically significant. WM and ML analysis obtained similar causal effects (Fig. 2). Additionally, using MR-PRESSO as a supplement to IVW, we obtained similar results (Fig. 4). In the sensitivity analysis, Cochran Q test yielded *P* = .13, and no heterogeneity was observed (Table 3); in addition, MR-Egger intercept analysis (intercept = 0.01; *P* = .23) demonstrated that no horizontal pleiotropy occurred (Table 3). By employing leave-one-out meta-analysis, the MR estimate was found to be reasonable (Fig. 5D).

### 3.5. Causal relationship between PBC and CD and UC

After selection, we included 8 and 14 SNPs for CD and UC, respectively. IVW analysis revealed that there was insufficient evidence for the causal effect of PBC on CD and UC (OR: 1.02, 95% CI: 0.98–1.07; OR: 1.00, 95% CI: 0.94–1.08; Fig. 2); WM, ML, and MR-Egger analysis revealed similar results. Using leave-one-out meta-analysis, it was found that the MR estimate was reasonable (Figs. 5E–F). Additionally, MR-Egger intercept analysis (intercept = −0.02, *P* = .44; intercept = 0.03, *P* = .27) demonstrated that no horizontal pleiotropy occurred (Table 3). The result of Cochran Q test showed no heterogeneity when testing the effect of PBC on CD but heterogeneity was observed when testing the effect of PBC on UC; however, this did not affect the results of the IVW analysis (Table 3).

## 4. Discussion

Using MR studies, we identified a positive causal relationship between PBC and IBD. Additionally, we found that while there is a positive correlation between CD and PBC, there is no reverse causality. UC exhibited no causal relationship with PBC

Zhu et al^[29]^ conducted an MR analysis on IBD and PBC and found an associationl between IBD and PBC, and found no association between UC and PBC. However, Zhao et al^[30]^ and Zhang et al^[31]^ found an associationl between UC and IBD (*P* = .02). Unlike the previous studies, we first selected a larger sample for the analysis, and then Bonferroni correction was performed on the *P*-value (*P* < .017) to make multiple comparisons; in contrast, Zhao et al and Zhang et al did not correct the *P*-value, which may have led them to draw different conclusions. However, after a literature review, Arai et al^[32]^ found that 2 cases of PBC were complicated by IBD during diagnosis and treatment, while Shizuma^[33]^ reported that patients with UC or CD were complicated by PBC. Therefore, although a certain cause-and-effect relationship between IBD and PBC exist, the relationship remains unclear.

Our findings demonstrate that a cause-and-effect relationship exists between IBD and PBC, indicating that they may share a common underlying pathogenesis. Studies have found that crosstalk between the liver and intestines may be a common mechanism leading to immune system damage in these organs.^[34–39]^ Early randomized controlled trials found that bacteria and bacterial products can enter the portal circulation through the damaged mucosa in IBD, leaving the liver continuously exposed to immunogenic stimuli.^[40,41]^ Conversely, bile acids secreted by the liver reach the intestine through the gut-liver axis and acutely regulate the intestinal immune system and intestinal microbiota.^[42–44]^ Activation of the immune and inflammatory responses can lead to the accumulation of antimicrobial reactive oxygen species, endoplasmic reticulum stress, and mitochondrial dysfunction.^[45,46]^

Some studies have found that the triangular relationship between bile acid-intestinal flora-cholestasis plays an important role in the pathogenesis of PBC.^[35]^ Future studies incorporating dedicated experimental approaches and molecular epidemiological investigations are warranted to elucidate the underlying biological mechanisms. Additionally, during leave-one-out meta-analysis in this study, we found specific SNPs that were significantly associated with the disease (IBD: rs9836291, rs2836883; UC: rs11209026, rs1801274; and CD: rs6651252, rs3197999). Studies have reported that the SNP (rs6651252) located on chromosome 8 can control the Wnt-responsive DNA enhancer element and control the expression of proto-oncogenes (MYC) in colon epithelial cells, thus promoting the occurrence of IBD.^[47]^ Our findings may provide new insights into the pathogenesis of IBD.

The causal relationship between IBD and PBC can help to improve disease prevention among patients with IBD and PBC. IBD cannot be overlooked in the diagnosis and treatment of PBC. According to the European Society of Liver Diseases PBC guidelines,^[48]^ the main tests included biochemical evidence of cholestasis (mainly elevated alkaline phosphatase/glutamyl transpeptidase); antimitochondrial antibodies (especially the M2 subtype) and other specific antinuclear autoantibodies (including anti-sp100 or gp210); magnetic resonance cholangiopancreatography and magnetic resonance elastography; and liver biopsy pathology. In addition to these diagnostic tests, video colonoscopies should be performed regularly. Cases of IBD related to PBC are still occasionally reported.^[11]^ Only timely detection and treatment of IBD during PBC can increase patient benefits.^[49]^ To prevent the occurrence of biliary malignant tumors in patients with IBD, measures should be taken to prevent and treat PBC in the early stage. Some studies have shown that in patients with IBD and biliary disease, treatment with ursodeoxycholic acid may reduce the occurrence of advanced cholangiocarcinoma.^[50]^ Therefore, patients with IBD require regular screening for PBC. The possible common pathogenic pathways of IBD and PBC can be studied by monitoring susceptibility genes that have a common impact on IBD and PBC.^[51]^ However, the underlying mechanisms of the association between IBD and PBC have been investigated in only a few studies; therefore, further research is needed to provide information on the prevention and early treatment of the disease.

Our study has certain advantages when compared with traditional research methods. First, this study followed a 2-sample bidirectional MR design, which reduces unobserved confounding that can distort the results of observational studies and some limitations of randomized controlled trials (representativeness and feasibility issues) in making causal inferences. Second, although patients with both IBD and PBC are rare, the study overcame the limitation of the number of patients. Nonetheless, some limitations should be noted. First, the genetic data used in this study were obtained from individuals of European ancestry. This population restriction may limit the applicability of the study results to other ethnic groups. Therefore, the results of this study are primarily applicable to individuals of European ancestry, and further research is needed to verify their generalizability to other ethnic groups. Future studies should be conducted in more diverse populations to validate and extend our findings. Second, overlapping samples may exist in exposure and outcome studies; however, estimating the extent of sample overlap is difficult. The potential bias from sample overlap was minimized in this study with an F statistic of > 10.^[52]^ Nevertheless, we recognize that undetected sample overlap could lead to weak instrumental bias, thereby affecting the accuracy of causal inference. Future studies should further optimize dataset selection to address this issue. Finally, some of the instrumental variables that were identified in this study were found to be statistically heterogeneous. Larger GWAS data are still needed to verify the conclusions obtained in this study through further MR.

## 5. Conclusion

This bidirectional, 2-sample MR study provides further insights into the association between IBD and PBC. Specifically, genetic prediction of CD is associated with an increased risk of PBC, suggesting a potential causal relationship. However, there is no evidence supporting a bidirectional genetic association between UC and PBC. These findings can significantly contribute to the advancement of the diagnosis and treatment of both IBD and PBC. Furthermore, our research provides novel insights into the complex interplay between IBD (UC and CD) and PBC.

## Acknowledgments

We would like to thank Editage (www.editage.cn) for English language editing.

## Author contributions

**Methodology:** Wenyuan Hong.

**Validation:** Xinxia Yang.

**Writing – original draft:** Zongchi Chen, Taiyong Fang.

**Writing – review & editing:** Weitao Hu, Taiyong Fang.
